# Proteomics of the dentate gyrus reveals semantic dementia specific molecular pathology

**DOI:** 10.1186/s40478-022-01499-1

**Published:** 2022-12-28

**Authors:** Merel O. Mol, Suzanne S. M. Miedema, Shamiram Melhem, Ka Wan Li, Frank Koopmans, Harro Seelaar, Kurt Gottmann, Volkmar Lessmann, Netherlands Brain Bank, August B. Smit, John C. van Swieten, Jeroen G. J. van Rooij

**Affiliations:** 1 Department of Neurology and Alzheimer Center Erasmus MC, Erasmus University Medical Center, Dr. Molewaterplein 40, 3015GD Rotterdam, The Netherlands; 2 Department of Molecular and Cellular Neurobiology, Center for Neurogenomics and Cognitive Research, Amsterdam Neuroscience, Vrije Universiteit Amsterdam, Amsterdam, The Netherlands; 3 Netherlands Institute for Neuroscience, Amsterdam, The Netherlands; 4 Institute of Physiology, Medical Faculty, Otto-Von-Guericke University, Magdeburg, Germany; 5 Department of Internal Medicine, Erasmus Medical Center, Rotterdam, The Netherlands; 6 Institute of Neuro- and Sensory Physiology, Medical Faculty, Heinrich Heine University, Düsseldorf, Germany

**Keywords:** Semantic dementia, Frontotemporal dementia, Frontotemporal lobar degeneration, TDP-43, Mass spectrometry, Proteomics, β-catenin, Cadherin-catenin complex

## Abstract

Semantic dementia (SD) is a clinical subtype of frontotemporal dementia consistent with the neuropathological diagnosis frontotemporal lobar degeneration (FTLD) TDP type C, with characteristic round TDP-43 protein inclusions in the dentate gyrus. Despite this striking clinicopathological concordance, the pathogenic mechanisms are largely unexplained forestalling the development of targeted therapeutics. To address this, we carried out laser capture microdissection of the dentate gyrus of 15 SD patients and 17 non-demented controls, and assessed relative protein abundance changes by label-free quantitative mass spectrometry. To identify SD specific proteins, we compared our results to eight other FTLD and Alzheimer’s disease (AD) proteomic datasets of cortical brain tissue, parallel with functional enrichment analyses and protein–protein interactions (PPI). Of the total 5,354 quantified proteins, 151 showed differential abundance in SD patients (adjusted *P*-value < 0.01). Seventy-nine proteins were considered potentially SD specific as these were not detected, or demonstrated insignificant or opposite change in FTLD/AD. Functional enrichment indicated an overrepresentation of pathways related to the immune response, metabolic processes, and cell-junction assembly. PPI analysis highlighted a cluster of interacting proteins associated with adherens junction and cadherin binding, the cadherin-catenin complex. Multiple proteins in this complex showed significant upregulation in SD, including β-catenin (CTNNB1), γ-catenin (JUP), and N-cadherin (CDH2), which were not observed in other neurodegenerative proteomic studies, and hence may resemble SD specific involvement. A trend of upregulation of all three proteins was observed by immunoblotting of whole hippocampus tissue, albeit only significant for *N*-cadherin. In summary, we discovered a specific increase of cell adhesion proteins in SD constituting the cadherin-catenin complex at the synaptic membrane, essential for synaptic signaling. Although further investigation and validation are warranted, we anticipate that these findings will help unravel the disease processes underlying SD.

**Supplementary Information:**

The online version contains supplementary material available at 10.1186/s40478-022-01499-1.

## Introduction

Semantic dementia (SD), also referred to as semantic variant of primary progressive aphasia (svPPA), is a clinical subtype of frontotemporal dementia (FTD) defined by impaired word comprehension and semantic memory [1–3]. Studies of its prevalence are limited, but is has been estimated that SD accounts for roughly 25–30% of all FTD patients [2]. Neuroimaging of SD patients typically reveals bilateral, but asymmetric atrophy of the anterior temporal lobes [4–6]. Unlike other forms of FTD, SD has a relatively slow disease progression and occurs nearly always sporadic [7]. Recently, our group identified somatic mutations in the gene *TARDBP*, encoding for TAR DNA Binding Protein 43 (TDP-43), as cause of disease in two patients [8]. Post mortem examination shows typical TDP-43 positive neuronal inclusions in the dentate gyrus of the hippocampus and long dystrophic neurites in the temporal cortex [9, 10]. This neuropathological entity, classified as frontotemporal lobar degeneration (FTLD) TDP type C, is consistently found in the majority of SD patients. The typical profile of cognitive, neuroimaging, and neuropathological features is suggestive for specific disease biology [11, 12], yet the pathophysiological mechanisms remain largely unexplored and therapeutic options are currently unavailable.

Over the past decade, mass spectrometry (MS) based methods have rapidly advanced and are now widely used to efficiently quantify thousands of proteins at once in selected cells or tissues of interest [13]. In the context of dementia, numerous studies used MS to analyze brain tissue, plasma, or cerebrospinal fluid [14, 15]. These studies contribute to an increased understanding of disease mechanisms and help to identify biomarkers and therapeutic targets. In contrast to the comprehensive histological characterization [10], relatively few studies applied MS on FTLD-TDP brain tissue [16]. Two studies identified abnormal protein abundances involving neuroinflammation, RNA processing, protein metabolism, and synaptic transmission [17, 18], and a recent report described the proteomic signatures and cell types involved in genetic FTLD [19]. One of the greatest challenges in brain tissue proteomics lies in the identification of specific disease processes, as the aforementioned pathways are typically observed in many different brain disorders and may represent coinciding neurodegenerative changes. Moreover, the variable approaches and disease subtypes challenge validation and solidification of the results, especially concerning FTLD as highly heterogeneous disorder. Comparison across datasets is therefore essential to differentiate processes broadly involved in neurodegeneration from disease specific alterations.

Here, we present the first quantitative proteomic study of SD, in which we assessed the relative protein abundance changes in the dentate gyrus. We compared our results with MS studies performed in other subtypes of FTLD and Alzheimer’s disease (AD), to separate SD specific from common neurodegenerative dysregulation. By discerning uniquely altered proteins and pathways, we aim to improve our understanding of the pathophysiological processes in SD, and to pave the way for the discovery of novel therapeutic targets.

## Materials and methods

### Patient tissue collection

**Fig. 1:**
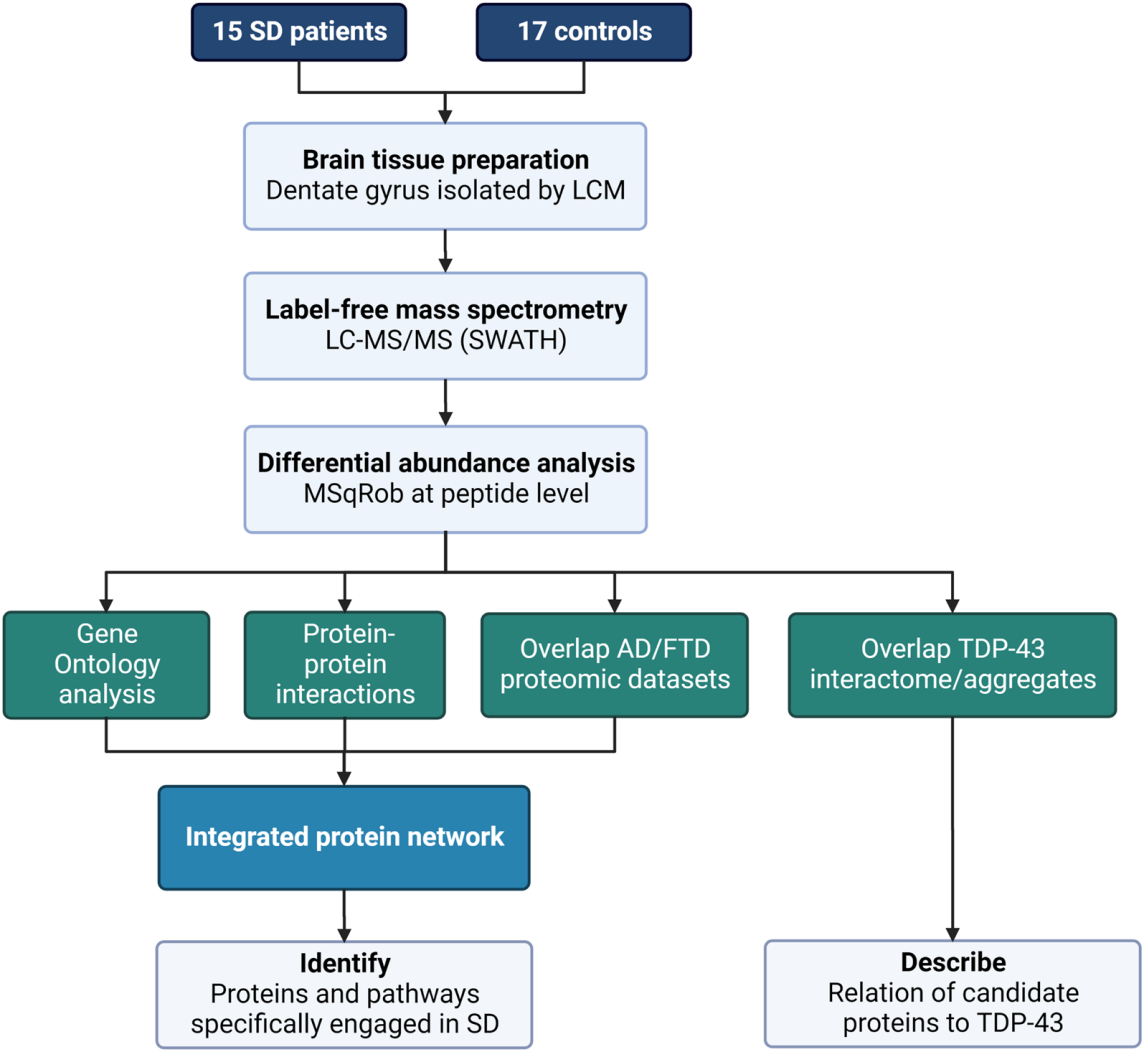
Workflow of the study. After brain tissue preparation of all 32 sample fractions, we performed mass spectrometry using SWATH (data-independent acquisition), followed by differential protein abundance analysis using MSqRob (quantitative protein-level statistical inference). The identified proteins with differential abundance in SD patients (*P*-value < 0.01 and ± 0.3 log2 fold change) were subjected to four parallel analyses (green boxes). The results of three analyses – Gene Ontology, protein–protein interactions, and comparison to other proteomic datasets – were integrated in a protein network, enabling collective interpretation of the main findings. Additionally, we compared the differential proteome of SD to proteins known to interact with TDP-43 and to proteins identified in TDP-43 neuronal aggregates based on previously published work (Laferrière et al., 2019). *Abbreviations*: SD = semantic dementia; LCM = laser capture microdissection; LC–MS/MS = Liquid chromatography with tandem mass spectrometry; AD = Alzheimer’s disease; FTD = frontotemporal dementia; TDP-43 = TAR DNA-binding protein 43

A schematic overview of the workflow is presented in Fig. 1. Patients with SD were ascertained from an ongoing clinical cohort study in the Netherlands, encompassing clinical and pathological data of FTD patients [20]. Fresh frozen brain samples of the hippocampus (left, except for two right-sided patient samples) were obtained from the Netherlands Brain Bank (NBB) of 15 patients with confirmed FTLD-TDP type C pathology [10], and 17 age and gender matched non-demented controls. The neuropathological reports of ten controls reported (mild) vascular/ischemic changes, though not observed in the hippocampi or temporal lobes. All patients were previously tested negative for pathogenic germline variants in the major FTD-related genes (*MAPT*, *GRN*, *C9orf72*, *TARDBP*, *TBK1*, *OPTN*, *SQSTM1*, *VCP*, *CHMP2B*, *FUS* [21]).

### Laser capture microdissection and protein separation

We selected the dentate gyrus as region of interest since it is characterized by abundant neuronal TDP-43 pathology, yet with limited tissue degeneration as compared to affected cortical areas. We performed laser-capture microdissection (LCM) using a Leica AS LMD system and equal volumes of dentate gyrus tissue (1.0 × 10^9^ μm^3^), followed by electrophoresis and in-gel digestion as previously described [22].

### Liquid chromatography and SWATH mass spectrometry

Peptides were analyzed by reverse phase liquid chromatography with tandem mass spectrometry (LC–MS/MS) using an Ultimate 3000 LC system and a TripleTOF 5600 mass spectrometer, set in data-independent acquisition (DIA) under the same parameters as reported previously [23]. Data were analyzed using the integrated software suite DIA-NN [24], an automated pipeline especially developed to process DIA data. The fasta protein sequence database provided as input was Uniprot human_UP000005640_9606. Deep learning was used to generate the in silico spectral library.

### Statistical analysis of differential protein abundance

MS-DAP version 1.0 (https://github.com/ftwkoopmans/msdap) was used for downstream analysis of the SWATH-MS output. Only peptides observed in at least 75% of all patient and control samples were selected. We evaluated principal components to visualize sample clustering, and the coefficient of variation as quality metric for reproducibility of replicate measurements within each sample group. After excluding evident outliers, peptide abundance values were normalized and MSqRob was used for differential testing at the peptide level, accounting for batches as random variable in the regression model [25]. A potential confounding effect of disease duration was evaluated using linear regression analyses on the subset of patient samples. *P*-values were adjusted for multiple testing using the Benjamini–Hochberg False Discovery Rate (FDR) procedure. Proteins with differential abundance in SD were defined by the thresholds of ± 0.3 log2 fold change (i.e., SD/control ratio > 1.35 or < 0.74) and adjusted *P*-value < 0.01.

### Gene ontology (GO) analysis

We performed functional enrichment analysis using g:Profiler (version e104_eg51_p15_3922dba) with all annotated human genes in the Ensembl database as background set [26]. In case of ambiguous protein annotations, only the first protein was included. The g:Profiler-based multiple testing correction (g:SCS method) was applied; terms with adjusted *P*-values < 0.05 were considered significant and selected for subsequent analyses. Upregulated and downregulated proteins were assessed separately. The following databases were examined: GO biological process (BP), GO cellular component (CC), and GO molecular function (MF). Since our objective was to distinguish specific disease processes, we narrowed the results to terms containing five up to 500 proteins to filter out broader parent terms typically designating more general, nonspecific pathways [27]. The top three nonredundant terms (i.e., at least 30% unique proteins) were prioritized for each GO category.

For an in-depth analysis of affected synaptic processes, we used SynGO (version: 20,210,225) [28], with FDR-based multiple testing correction and brain expressed proteins as background. SynGO enrichment analysis was performed on cellular components (CC) and biological processes (BP) ontology terms.

### Protein–protein interactions

We extracted known protein–protein interactions (PPI) between all proteins with differential abundance in SD from the STRING database (Search Tool for the Retrieval of Interacting Genes/Proteins) based on experimentally determined interactions, phylogenetic co-occurrence, and co-expression [29]. The minimum required confidence score was set to 0.4 (medium confidence).

### Comparison to FTLD and AD proteomic changes

To identify proteins potentially unique to SD, we compared our results to previous MS studies investigating TDP-43 and AD pathology. The literature was searched using the following terms: (‘proteomic*’ OR ‘mass spectrometry’) AND (‘frontotemporal dementia’ OR ‘frontotemporal lobar degeneration’ OR ‘Alzheimer’s disease’). The resulting articles were manually filtered to meet the following criteria: 1) quantitative MS study conducted on brain tissue of FTLD-TDP or AD patients compared to non-demented controls; 2) hippocampus, temporal cortex, or frontal cortex tissue; 3) sample size > 5 cases; and 4) full list of differentially expressed proteins available. Non-human studies or studies without control group were excluded, as well as studies with overlapping patient cohorts. We extracted lists of all quantified and differentially expressed proteins for comparison to our dataset.

### Comparison to TDP-43 interactome and aggregates

As TDP-43 is the major disease protein, we aimed to determine possible overlap across the proteins altered in SD patients and the TDP-43 interactome. We extracted all known first shell protein interactors of TDP-43 from STRING, applying the same settings as described above. The proteins found to directly interact with TDP-43 were compared to the differentially expressed proteins in SD. Additionally, we evaluated the overlap between our results and those of the MS study by Laferrière et al. investigating the insoluble proteome of different FTLD-TDP subtypes, following biochemical isolation of pathological TDP-43 aggregates from cortical brain tissue [30].

### Immunoblotting

Following the analyses as described above and based on availability of validated antibodies, a selection of candidate proteins was prioritized for immunoblotting. We used fresh frozen whole hippocampal tissues, because laser-captured dentate gyrus tissue was not available for immunoblotting. Two control samples were excluded because the tissue section did not include the whole dentate gyrus (NDC9), or because of insufficient tissue available for all blots (NDC10). RIPA buffer containing protease and phosphatase inhibitors were added to the tissues which were lysed using the Genie disruptor. The subsequent whole tissue lysates were used, without centrifuging, to be most consistent with the tissue samples selected for MS. Proteins were denatured and separated by SDS-PAGE using Criterion™ precast gels (Bio-Rad) and transferred onto a PVDF membrane (N-cadherin) or a nitrocellulose (β-catenin and γ-catenin). Membranes were blocked with 5% non-fat milk, incubated with primary antibody overnight at 4 °C and then with matching fluorescent secondary antibodies (IRDye, LI-COR) for 1 h at RT. After washing, membranes were scanned using the Odyssey DLx system (LI-COR Bioscience). Images were quantified using Image Studio Lite software (version 2.0.38). Differences in loading were corrected using the housekeeping gene GAPDH. Immunoblot signals were normalized to the mean of the control samples. We used the following primary antibodies: anti-β-catenin (1/400, Santa Cruz, sc-7963), anti-γ-catenin (1/500, Cell signaling, #2309), and anti-N-cadherin (1/400, C32, BD Biosciences).

### Immunohistochemistry

Routine immunohistochemistry was carried out by the NBB. We performed additional staining on dentate gyrus paraffin-embedded tissue from all included patients and a random subset of three non-demented controls. The following antibodies were used: anti-β-catenin (1/1000, Santa Cruz, sc-7963), and anti-N-cadherin (1/800, Abcam, ab18203).

## Results

### Protein quantification and differential protein abundance in SD

To interrogate quantitative proteomic changes, we performed label-free DIA mass spectrometry on the dentate gyrus of 15 SD patients with confirmed FTLD-TDP type C pathology and 17 non-demented control subjects (Table 1). This allowed quantification of 37,465 peptides per sample on average (range 30,046–43,282). Principal component analyses revealed two controls and one patient sample as clear outliers (Additional file 1: Fig. S1). For one sample (NDC9), this could be explained because the tissue section did not include the whole dentate gyrus. After removal of these three samples, a mean coefficient of variation of ~ 30% for peptide quantification indicated high reproducibility between replicate samples in both groups (Additional file 1: Fig. S2). Following quality filtering (see methods), 28,499 peptides were included in the differential abundance analysis, mapping to 5,354 proteins across the 29 samples. Linear regression analyses indicated that disease duration has no significant effect on protein abundance values among patients. All statistical results are available in Additional file 2: Table S1.

**Table 1:** Demographic, clinical, and post-mortem characterisitcs of selected semantic dementia patients and non-demented controls

Case type	Sample number	Sex	Age at death	Disease duration (yrs)	Post mortem delay (min)	Braak stage	Thal stage	Batch number
Semantic dementia	SD02	M	62	14	280	0	0	2
	SD04*	F	65	20	330	0	0	3
	SD05	M	66	10	320	0	0	3
	SD07	F	64	11	385	0	0	2
	SD08	M	69	12	320	0	0	1
	SD09	F	74	11	240	1	0	1
	SD10	M	68	13	420	1	3	1
	SD11	M	66	15	345	1	0	2
	SD12	F	72	12	375	2	2	1
	SD13	F	72	9	250	3	2	4
	SD14	M	72	15	455	2	1	4
	SD16	M	74	13	225	0	1	2
	SD17	F	67	9	275	2	1	3
	SD18	F	68	16	225	0	0	3
	SD19	M	61	12	290	0	0	4
*n* = 15	Average		68	13	316			
Controls	NDC1	M	64		505	1	1	1
	NDC2	M	75		430	1	1	1
	NDC3	F	57		460	0	0	1
	NDC4	F	90		350	2	0	1
	NDC5	F	69		510	1	0	2
	NDC-6	F	54		335	0	0	2
	NDC-7	M	70		1245	1	0	2
	NDC8	F	68		630	2	1	2
	NDC9*	F	74		360	0	0	3
	NDC10*	M	69		325	1	1	3
	NDC11	F	92		265	3	1	3
	NDC12	M	79		345	2	1	3
	NDC13	F	79		840	3	2	4
	NDC14	F	67		790	0	0	4
	NDC15	M	56		300	3	2	4
	NDC16	F	75		550	1	1	4
	NDC17	M	71		345	1	2	4
*n* = 17	Average		71	NA	505			

For the mass spectrometry, samples were split in four batches of each 3–5 patient and control samples. All patients were characterized by FTLD-TDP type C neuropathology

*SD* semantic dementia, *NDC* non-demented control

*Three samples from batch 3 (one patient and two control samples) were excluded from differential abundance analysis following quality control, indicating these samples as outliers. Note that the samples partially overlap with those reported in a previous publication on somatic mutations in semantic dementia[8]

**Fig. 2:**
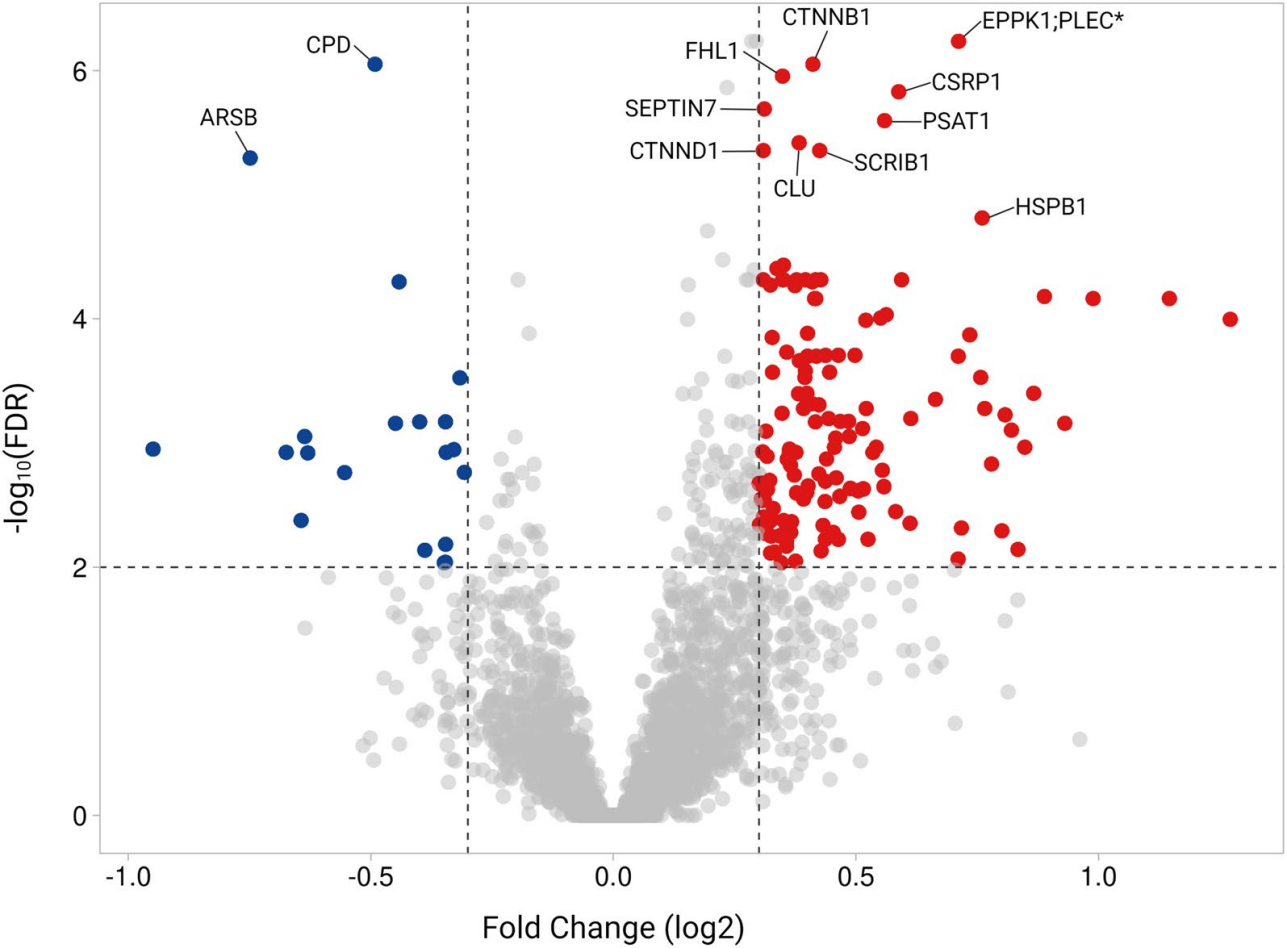
Proteins with differential abundance in SD patients compared to non-demented controls. Fold-changes (x-axis) were determined and *P*-values (y-axis) adjusted for multiple testing using the FDR approach (Benjamini–Hochberg procedure). Proteins with differential abundance in SD were defined by adjusted *P*-value < 0.01 and log2 fold change threshold of ± 0.3, resulting in 131 upregulated and 20 downregulated proteins in SD patients. All statistical results are available in Additional file 2: Table S1 and Additional file 2: Table S2. *Ambiguous protein annotation; in such cases only the first protein was included in subsequent analyses

The differential proteomic signature was composed of 151 unique proteins in SD patients as compared to controls (adjusted *P*-value < 0.01 and ± 0.3 log2 fold change), of which 131 were upregulated and 20 downregulated (Fig. 2; Additional file 2: Table S2). The top 20 proteins with significant differential abundance (all upregulated) in patients are shown in Table 2. This list indicates a variety of affected cellular functions, i.e., cytoskeletal organization (EPPK1, SEPTIN7), cell adhesion (CTNNB1, CTNND1), neuronal morphogenesis (CSRP1, SCRIB), metabolic/catabolic processes (HIBADH, ADH5, AK1), and proteasomal degradation (CLU, ARSB, HSPB1).

**Table 2:** The top 25 proteins with significant differential abundance in semantic dementia patients versus controls

Gene symbol	Protein name	Fold change	Adjusted *P*-value
EPPK1	Epiplakin	2.04	5.80e-07
CPD	Carboxypeptidase D	0.61	8.87e-07
CTNNB1	Catenin beta-1	1.51	8.87e-07
FHL1	Four and a half LIM domains protein 1	1.42	1.11e-06
CSRP1	Cysteine and glycine-rich protein 1	1.80	1.48e-06
SEPTIN7	Septin-7	1.36	2.03e-06
PSAT1	Phosphoserine aminotransferase	1.75	2.53e-06
SCRIB	Protein scribble homolog	1.47	3.82e-06
CLU	Clusterin	1.53	4.41e-06
CTNND1	Catenin delta-1	1.36	4.41e-06
ARSB	Arylsulfatase B	0.47	5.06e-06
HSPB1	Heat shock protein family b (small) member 1	2.14	1.54e-05
HIBADH	3-hydroxyisobutyrate dehydrogenase	1.42	3.70e-05
RAB12	Ras-related protein Rab-12	1.40	3.93e-05
ADH5	Alcohol dehydrogenase class-3	1.49	4.85e-05
AK1	Adenylate kinase isoenzyme 1	1.42	4.85e-05
CAMK2D	Calcium/calmodulin-dependent protein kinase type II subunit delta	1.52	4.85e-05
CFDP1	Craniofacial development protein 1	1.81	4.85e-05
ESD	S-formylglutathione hydrolase/Esterase D	1.36	4.85e-05
GNAI2	Guanine nucleotide-binding protein G(I) subunit alpha-2	1.42	4.85e-05

The top 20 proteins with significant differential abundance (adjusted *P*-value < 0.01) are listed for semantic dementia patients versus controls

### Gene ontology (GO) analysis

GO analysis of biological processes (BP) with all 151 proteins as input revealed an enrichment of immune response activation, gliogenesis, and cell junction assembly in the upregulated proteins, whereas various proteins associated with catabolic and metabolic processes were downregulated (Table 3). Assessment of cellular components (CC) yielded the most results, with terms related to cellular adhesion (i.e., cell-substrate junction, adherens junction) enriched for upregulated proteins, whereas downregulated proteins showed enrichment for the lysosome. Cadherin binding was most strongly enriched in the category molecular functions (MF), followed by terms associated with catalytic activity (i.e., GTPase activity and oxidoreductase activity). Given the smaller proportion of downregulated proteins (*n* = 20), these are less represented in all domains than upregulated changes (Table 3). A complete overview of all terms with corresponding proteins and statistical output is provided in Additional file 2: Table S3 and Additional file 2: Table S4.

**Table 3:** Gene Ontology analysis showing the top 10 enriched terms for each category

Directional change	Term name	Intersection size^a^	Adjusted *P*-value
Biological processes			
Up	neutrophil degranulation*	18/483	2.29e-05
Up	neutrophil activation involved in immune response	18/488	2.69e-05
Up	neutrophil mediated immunity	18/500	3.90e-05
Down	glycos-amino-glycan catabolic process*	4/61	5.72e-04
Down	amino-glycan catabolic process	4/67	8.37e-04
Up	gliogenesis*	12/318	6.31e-03
Up	cell junction assembly	14/442	7.24e-03
Down	glycos-amino-glycan metabolic process	4/159	2.64e-02
Up	regulation of trans-synaptic signaling	13/435	3.03e-02
Down	sulfur compound catabolic process	3/54	3.29e-02
Cellular components			
Up	cell-substrate junction*	24/427	5.51e-13
Up	focal adhesion	23/420	3.99e-12
Down	lysosomal lumen*	7/97	9.81e-10
Down	vacuolar lumen	7/174	6.18e-08
Up	adherens junction*	11/171	9.53e-06
Up	extrinsic component of plasma membrane	11/178	1.44e-05
Up	cell–cell junction	17/496	1.83e-05
Up	basal plasma membrane	12/250	5.51e-05
Up	ficolin-1-rich granule lumen	9/124	6.60e-05
Down	primary lysosome	5/154	8.45e-05
Molecular functions			
Up	cadherin binding*	20/333	9.37e-11
Up	GTPase activity*	12/300	7.00e-04
Up	oxidoreductase activity [1]*	8/128	1.75e-03
Up	disordered domain specific binding	5/34	1.99e-03
Up	oxidoreductase activity [2]	8/139	3.23e-03
Up	pyrophosphatase activity	14/482	3.80e-03
Up	nucleoside-triphosphatase activity	13/424	4.64e-03
Up	hydrolase activity [1]	14/493	4.90e-03
Up	hydrolase activity [2]	14/494	5.02e-03
Up	GTP binding	12/381	8.04e-03

The three classical GO categories were assessed for upregulated and downregulated proteins separately, using g: Profiler with all annotated human genes as background and significance threshold set to 0.05. The results were filtered to terms containing five up to 500 proteins, yielding a total of 13 terms for biological processes (A), 20 for molecular functions (B), and 52 for cellular components (C). A complete overview of all terms with corresponding proteins and statistical output is provided in Additional file 2: Table S3 and Additional file 2: Table S4

*The top three nonredundant terms of each GO category were prioritized for further analysis

^a^Indicates overlap between input proteins (dysregulated in SD patients) and all proteins of the respective GO term

Detailed analysis of synapse enriched proteins using SynGO, indicated that 36/151 deregulated proteins could be mapped to unique synaptic annotated genes. Ten significantly enriched CC terms and three BP terms indicated involvement of both presynaptic and postsynaptic compartments and functions (Additional file 1: Fig. S3 and Additional file 2: Table S5). The CC terms included the more specific annotations (third/fourth level in hierarchical structure) ‘presynaptic active zone, cytoplasmic component’ and ‘postsynaptic density, intracellular component’ due to the genes CTNNA2, CTNNB1, and CTNND1.

### Protein–protein interactions (PPIs)

**Fig. 3:**
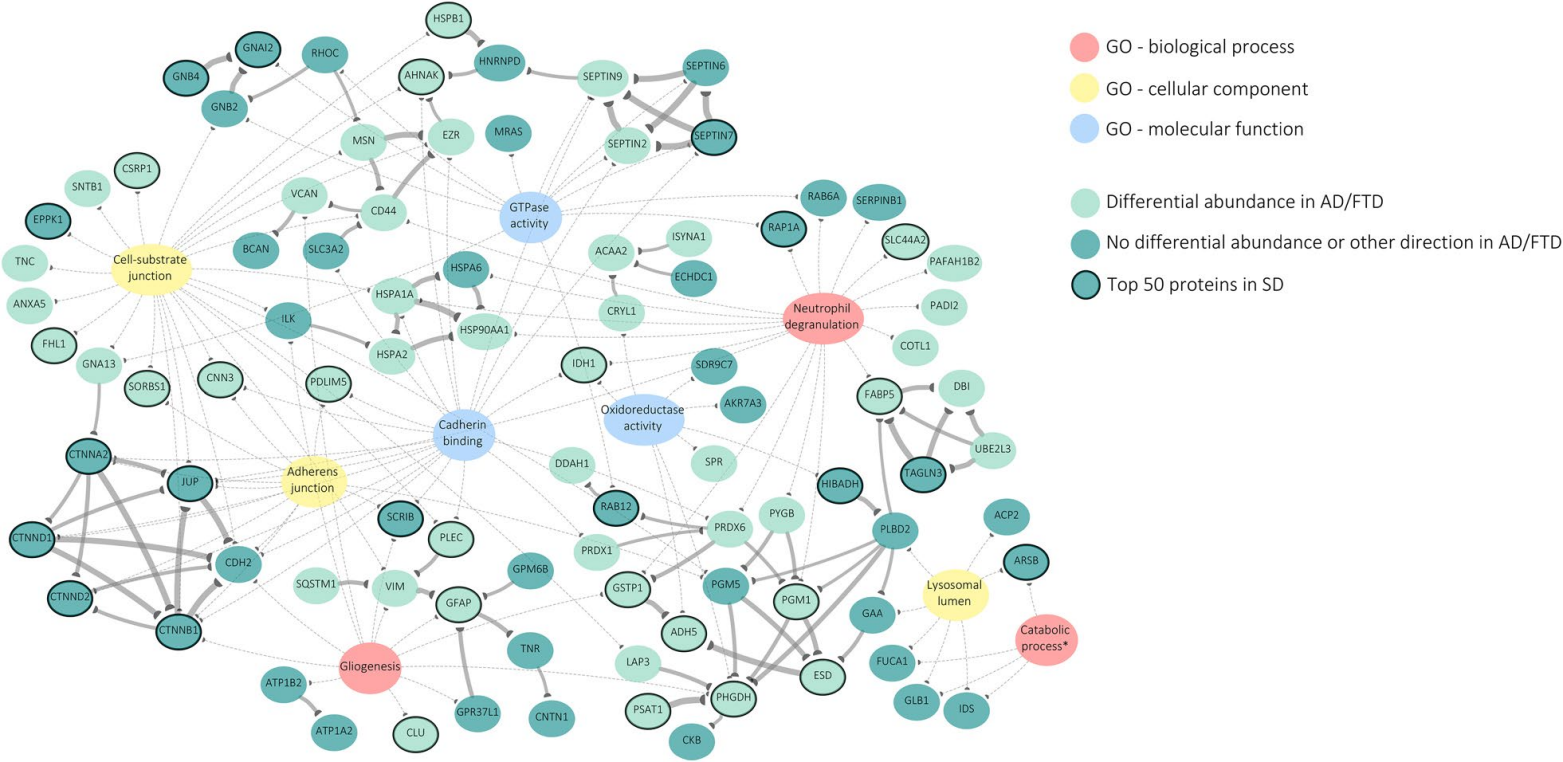
Integrated protein interaction network of the different parallel analyses. Ninety-two out of 151 differentially expressed proteins in SD are depicted in this figure, following their association (thin edges) with the top three GO enriched terms for each category (BP/MF/CC), and the protein–protein interactions (PPIs) derived from STRING database (thickness of edge according to interaction score). The remaining 59 proteins can be found in Additional file 2: Table S2. All GO terms include upregulated proteins, except for lysosomal lumen (CC) and catabolic process (BP). The nodes are colored according to the comparison to previously published proteomic datasets of FTLD and AD patients. This indicates half of the proteins depicted in this figure as potentially unique to SD (dark colored nodes), whereas the remainder (*n* = 48) was differentially expressed in the same direction in at least one of the FTLD/AD studies (light colored nodes). The 50 most strongly dysregulated proteins in SD are highlighted by thick border. A cluster of catenin and cadherin proteins – in the lower left corner – particularly stands out based on the PPIs, association with the GO terms adherens junction (CC) and cadherin binding (MF), and strong upregulation in SD (five out of six proteins in top 50). *The term ‘catabolic process’ was shortened from ‘glycos-amino-glycan catabolic process’. Abbreviations: GO = gene ontology; FTD = frontotemporal dementia; AD = Alzheimer’s disease; SD = semantic dementia

Amongst all proteins, we evaluated known PPIs using the STRING database. In total, 68/151 proteins were found to interact with at least one other protein of the 151 (PPI enrichment *P*-value < 1.0e-16), including six larger clusters consisting of at least four proteins (Additional file 1: Fig. S4). The corresponding interaction scores are provided in Additional file 2: Table S6. In Fig. 3, we visualized these interactions in a network integrated with the top three GO enriched terms for each category (BP/MF/CC). The figure indicates several associations between the PPI clusters and GO terms. In particular three larger clusters of interacting proteins can be distinguished, associated with (combinations of) the following GO terms: (1) cell-substrate junction [CC] and GTPase activity [MF]; (2) adherens junction [CC] and cadherin binding [MF]; and (3) neutrophil degranulation [BP].

### Comparison to FTLD and AD proteomes

To identify proteins potentially unique to SD/FTLD-TDP type C, we compared our results to eight previously published proteomic datasets of FTLD-TDP (*n* = 3) and AD (*n* = 5) brain tissue. The FTLD-TDP studies included TDP-subtypes A, B, and unspecified cases. Detailed characteristics of the studies are presented in Additional file 2: Table S7. Seventy-two out of 151 proteins with differential abundance in SD showed the same direction of dysregulation in ≥ 1 other study, implying alterations common to neurodegeneration. Fifty proteins were found altered only in SD, while another nine were not detected by any of the eight studies. The remaining 20 proteins (20/151, 13%) showed significant changes in the opposite direction in AD/FTLD as compared to SD, mostly due to downregulation in AD while being upregulated in SD (see Additional file 1: Fig. S5 and Additional file 2: Table S8 for more details). The *P*-values of these 20 proteins were evenly dispersed throughout the total set, lowering the possibility that these all represent false positives. To evaluate if this discordance also occurs between the other FTLD/AD studies, we performed the same comparison using each of those published results as input list, and detected similar fractions of proteins with discordant direction between studies (range 4–17%; average 8.5% of all significant proteins per study). This led us to conclude that the 20 discordant proteins may still include relevant findings and should be taken along in subsequent analyses.

Altogether, 79 proteins can be marked as potentially uniquely altered in SD (see Additional file 2: Table S8 for complete list). Highlighting these proteins in Fig. 3 indicates that the PPI cluster of six proteins related to adherens junction/cadherin binding shows most specificity for SD. Five of these proteins—CTNNB1, CTNND1, CTNNA2, JUP, and CDH2—are part of a specific cellular component, the cadherin-catenin complex. All six are significantly upregulated in SD with similar fold change (1.4–1.6), and five out of six are listed in the top 50 (Additional file 2: Table S2). Of the proteins outside the PPI clusters, those implicated with the lysosomal lumen also show potential SD specificity.

### Immunoblotting of catenin and cadherin proteins

**Fig. 4:**
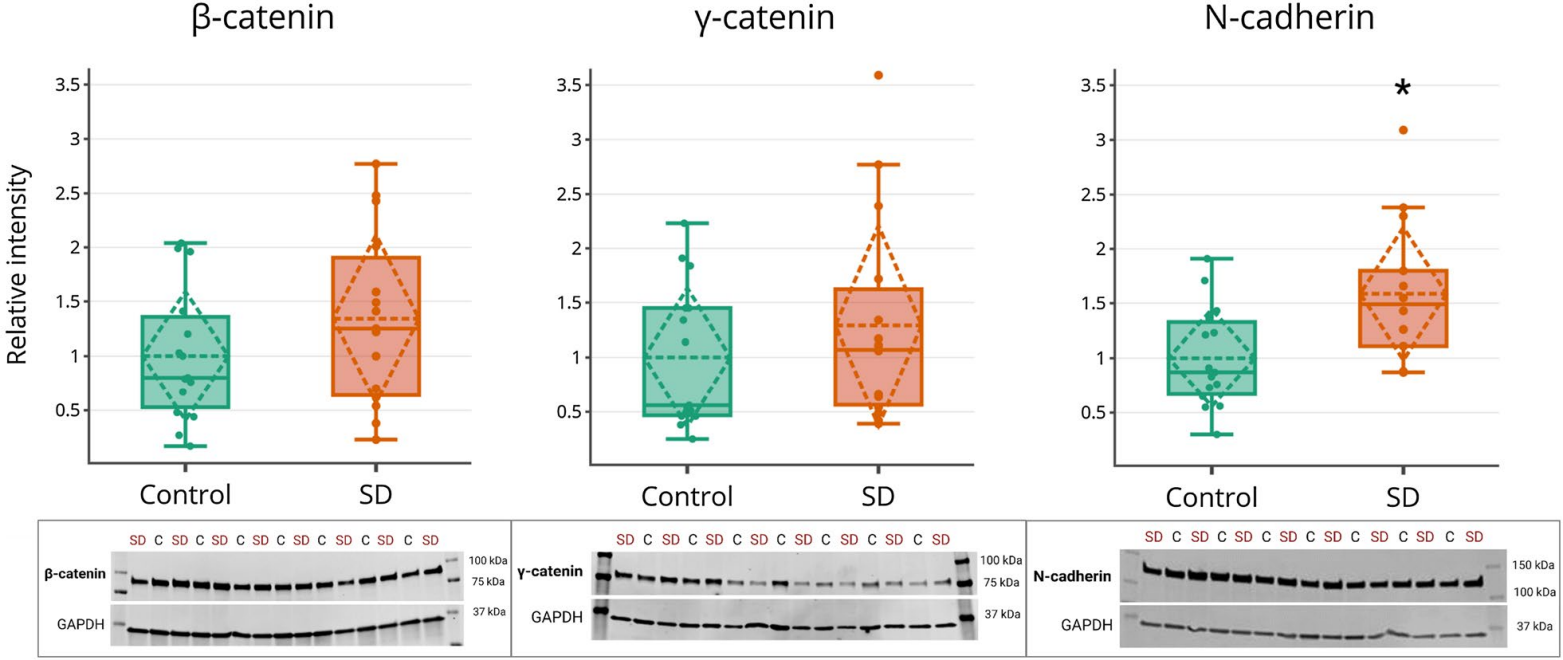
Quantified immunoblots of three proteins related to the cadherin-catenin complex. Immunoblotting was performed using whole hippocampal tissue of 15 SD patients and 15 non-demented controls. β-catenin/CTNNB1 was insignificantly increased in SD patients versus controls (FC 1.34, *P*-value 0.20). Immunoblotting of γ-catenin/JUP showed a similar trend (FC 1.29, *P*-value 0.34). For N-cadherin/CDH2, 14 SD samples were included in the analysis (as GAPDH immunoblot failed for one sample [#SD12]), revealing a significant difference between patients and controls (FC 1.59, *P*-value 0.01). Original images of one immunoblot gel are shown below the respective boxplots, with SD = semantic dementia patients, and C = controls. An overview of all immunoblots is provided in Additional file 1: Fig. S5, and all quantified data can be found in Additional file 2: Table S9

As internal validity of our label-free MS approach, we performed immunoblotting on whole hippocampal tissue of three upregulated proteins related to the cadherin-catenin complex (i.e., CTNNB1, JUP, and CDH2) in 15 SD patients and 15 controls from our initial MS cohort. The results showed an increased signal for CTNNB1/β-catenin (FC 1.34, *P*-value 0.20) and JUP/γ-catenin (FC 1.29, *P*-value 0.34) in SD patients versus controls, albeit not significant (Fig. 4). Immunoblotting of CDH2/N-cadherin showed a significant increase in cases compared to controls, (FC 1.59, *P*-value 0.01). The original blots are provided in Additional file 1: Fig. S6, and the quantified data in Additional file 2: Table S9.

### Immunohistochemistry of cadherin-catenin proteins

To localize the upregulation of catenins in the dentate gyrus, we performed immunostaining on hippocampal paraffin-embedded tissues of all included SD patients and a random subset of three non-demented controls. Anti-β-catenin staining resulted in diffuse background staining, not different to controls (data not shown). As γ-catenin is homologous to β-catenin, staining of this protein was not performed. In four out of the 15 patients, immunohistochemistry using N-cadherin antibody showed robust irregular cytoplasmic staining of the dentate gyrus’ granular cells with some neurons more diffusely stained, and others with a pretangle-like aspect (Additional file 1: Fig. S7). These cases with increased staining did not correlate to those with the highest protein abundances as measured by MS.

### The proteomic profile of SD in relation to the TDP-43 interactome

As final analysis, we sought to investigate a potential link between the identified deregulated proteins in SD and the TDP-43 interactome; since pathological TDP-43 is the major disease protein in SD. We extracted all first shell interactors of TDP-43 from STRING, resulting in 131 interactions (Additional file 2: Table S10). Only a single protein, HSP90AA1, was found to overlap between the TDP-43 interactome and the 151 proteins altered in SD. Additionally, comparison with 18 inclusion proteins recently identified in FTLD-TDP subtype C [30] showed that only one of these proteins, IDH1, was present in our dataset.

## Discussion

This study describes the altered proteome of the dentate gyrus in SD patients to obtain more insight into disease specific mechanisms. Amongst the dysregulated proteins, we distinguished a cluster of interacting proteins constituting the core component of a cell adhesion complex at the synaptic junction, referred to as the cadherin-catenin complex. The upregulation of these proteins might represent SD specific modifications, playing an important role in the pathophysiologic cascade of FTLD-TDP type C pathology.

### Integrating different analyses points towards SD specific alterations

Functional enrichment analysis of the 151 differentially expressed proteins in SD indicated changes also previously reported in cortical brain regions affected by TDP-43 and AD pathology such as immune response activation, astrogliosis, cellular adhesion, and metabolic processes [17–19, 31, 32], likely reflecting general neurodegenerative changes. The identification of these established pathways confirms that our proteomic strategy detects the global processes affected or caused by neuronal loss. Most of the preceding studies were performed on cortical brain tissue affected by severe atrophy. In contrast, we selected the dentate gyrus which shows abundant TDP-43 pathology in the absence of severe neuronal loss, thus, more likely to constitute specific dysregulation instead of merely common neurodegeneration.

To pinpoint disease specific alterations, we integrated the results following Gene Ontology analysis, protein–protein interactions, and comparison to proteomic changes in the brains of FTLD and AD patients. A cluster of six biologically linked and interacting proteins – five catenins and one cadherin – were not previously reported as upregulated in FTLD or AD. Two proteins (δ-catenin 1 and 2) were observed downregulated in two AD studies [33, 34]. These proteins not only stood out due to strong upregulation in SD, but also because of their association with a specific cellular component, the cadherin-catenin complex. SynGO analysis additionally revealed their enrichment and relationship to the synaptic membrane, suggesting specific synaptic changes attributable to this protein complex.

With ‘cell adhesion’ as parent GO term, we evaluated whether proteins related to other major cell adhesion complexes (e.g., neuroligin and neurexin [35]) were also affected in patients, but this was not the case.

By additional comparison to the TDP-43 interactome and a recent proteomic study of insoluble TDP-43 aggregates, we confirmed that the observed proteomic profile of SD does not represent TDP-43 binding partners nor its co-aggregated inclusion proteins. This is consistent with our method, as we did not follow specific procedures to capture this fraction of proteins. Instead, we focused on the global cellular changes in mostly soluble proteins in the affected neurons to understand the broad nature of impaired protein homeostasis, preceding or following protein aggregation.

### Immunoblotting of cadherin-catenin proteins in SD

Immunoblotting of N-cadherin (CDH2) blots demonstrated a significant increase in patients compared to control samples. For β-catenin (CTNNB1) and γ-catenin (JUP), we observed a trend of upregulation in SD. The difference with controls was not significant, although fold changes were similar as observed with MS. As laser-captured dentate gyrus (selected for MS) was not available for immunoblotting, we used whole hippocampal tissue which possibly increased the variation amongst the samples. It is also plausible that MS is a more sensitive technology with quantification of digested peptides, in contrast to immunoblotting which only captures full length solubilized proteins. Notwithstanding the need for further validation, we cannot exclude that the cadherin-catenin proteins plays a specific role in the SD dentate gyrus, given the fact that we detected multiple members of the complex [36], all measured by MS with similar enrichment in patients (fold change ~ 1.5).

### The role of cadherin-catenin complex proteins in cell adhesion and synaptic functioning

The cadherin-catenin complex plays an important role in cell adhesion at the synaptic junction [37], with β-catenin and N-cadherin as key regulating components, and α-catenins and δ-catenins as important binding partners in linking the complex to the actin cytoskeleton [38–43]. Gamma-catenin, also called junction plakoglobin, is homologous to β-catenin and may replace its function in the complex [36]. Aberrant functioning and mutations in independent cadherin and catenin family members have been associated with several disorders such as AD, autism, and intellectual disability [43]. However, increased abundance of these proteins has not been described previously in relation to neurological disease.

The most robust signal with MS was found for β-catenin. Besides its involvement in cell adhesion, β-catenin is known as transcription factor, and abnormal β-catenin/Wnt signaling has been implicated in many brain pathologies such as AD and brain cancers [44–46]. Activation of Wnt signaling has been observed in progranulin deficiency-linked FTLD-TDP, with increased nuclear localization of β-catenin [47]. Based on our results, several aspects oppose against a mechanism solely driven by altered Wnt signaling in SD. First, we detected multiple cadherin and catenin proteins not involved in Wnt signaling. Second, important targets of β-catenin (e.g., LEF1 and TCF7L2) or regulators of its activity (e.g., GSK3A and CK1) [48] were not altered. Finally, we did not observe increased nuclear β-catenin staining with immunohistochemistry (not shown). Altogether, we suggest that the upregulation of β-catenin in SD is related to its role in the cadherin-catenin complex, implicated in cell adhesion and synaptogenesis.

### The cadherin-catenin complex changes in relation to TDP-43 pathology

Although TDP-43 has been associated with synaptic functioning [49], a direct link between TDP-43 and synaptic cell adhesion has thus far not been established. Nonetheless, the importance of cell adhesion proteins for synaptic functioning has been widely investigated in the context of neurodegeneration, as the deposition of pathological proteins (e.g., Aβ plaques and tau aggregates) may induce synapse destabilization via changes in synaptic cell adhesion molecules [50–52]. In models of both AD and FTLD, abnormalities in synaptic stability have been shown to occur in early stages of disease, with more extensive synaptic damage occurring during the course of disease progression [53, 54].

Via enhanced association with N-cadherin, β-catenin stabilizes N-cadherin function to improve spine stability and synaptic transmission [55]. Dynamic changes of synaptic adhesion have been demonstrated by increased synaptic N-cadherin in response to loss of other cell adhesion molecules [56]. A study of spine dynamics demonstrated that acute disruption of N-cadherin leads to an initial compensatory attempt by the cell via an increase of β-catenin and increased binding between β-catenin, α-catenin, and δ-catenin [40]. A similar cascade of events might occur in SD, in which upregulation of the cadherin-catenin complex serves to enhance synaptic stability. However, persistence of highly stable synapses compromises structural plasticity, which is essential for synaptic network reorganization and learning related processes, especially in the hippocampus [57]. The stabilizing effect of this compensatory mechanisms might also be limited when the toxicity of pathological TDP-43 increases during the course of disease progression, leading to overt synaptic loss. More in-depth investigation is required to provide insight into the precise molecular mechanisms.

### Possible future steps to further characterize the cadherin-catenin complex in SD

Foremost, the upregulation of proteins related to the cadherin-catenin pathway in SD warrants validation; for instance by characterization in additional patient groups and brain regions. A possible next step is to localize the upregulation of β-catenin, N-cadherin, and other binding partners to specific cells and/or subcellular compartments. Staining of β-catenin did not reveal evident disparities in SD patients compared to controls, despite this protein being most strongly altered in both MS and immunoblotting. This discrepancy could be due to the properties of the different antibodies used. As for N-cadherin, immunohistochemistry did not reveal consistent changes among patients, although we did observe a unique staining pattern in several cases of which the underlying cause needs to be clarified. Applying other techniques (e.g., immunofluorescence, immunoprecipitation), and possibly more advanced technologies such as spatial proteomics [58], will hopefully shed more light on the functions and localizations of catenins and cadherins in SD. Studying signal transduction and synapse dynamics in cellular and/or animal models would also provide important mechanistic insight into the role of the cadherin-catenin complex in synaptic plasticity.

### Strengths and limitations

We performed the first proteomic study specifically analyzing SD/FTLD-TDP type C neuropathology. The isolated dentate gyrus represents a unique brain region, as it contains profound TDP-43 pathology with limited neuronal loss. Our sample size of 15 patients is substantial considering the rarity of the disorder. To pinpoint disease specific pathways, we integrated different in silico analyses and compared our findings to previous proteomic studies of other dementia subtypes. An important first consideration is the variety amongst these studies regarding phenotypes, brain regions, and analytical approaches. This was illustrated by the observation of proteins with discordant directional change. It emphasizes the need to replicate our results, at present hindered by the absence of other SD proteomic datasets. Second, for two patients only the right hemispheres were available for mass spectrometry, as opposed to the left for all other subjects. This might have influenced our results as the left temporal lobes were more severely affected, consistent with left-lateralized SD. Another limitation of this study is its focus on the dentate gyrus only, as the temporal lobes are also severely affected in SD, and characterized by neuronal inclusions distinct from those in the hippocampus. Proteomic analysis of the temporal cortex of our patients is currently ongoing to enable a comparative analysis of both regions, and to provide a more complete representation of the neuropathological changes in SD. This comparison will also give insight into whether the newly identified role of cadherin and catenin proteins in the SD proteome is seen across differently affected brain regions. Finally, by focusing on a strict selection of candidates, we may have overlooked other proteins also of interest for the biology of SD. An expansion of our current analyses could be worthwhile in future work.

## Conclusion

This work provides a description of the proteomic changes in the dentate gyrus of SD patients, including dysregulation of pathways related to the immune response, metabolic processes, and cell adhesion. Integrated analyses of functional enrichment, protein interactions, and comparison to other proteomic datasets highlighted a cluster of interacting proteins constituting the cadherin-catenin complex, implicated in synaptic cell adhesion. Further validation, replication of our findings, and comparative studies across different brain regions and FTLD subtypes are required to corroborate altered dynamics of these proteins in SD, as well as functional work to elucidate a possible interplay with pathological TDP-43 aggregate formation. We anticipate that this will result in a deeper understanding of the complex molecular changes in this severe disorder.

## Supplementary Information

**Additional file 1**. **Table S1**. List of all quantified proteins following mass spectrometry of the dentate gyrus in SD patients (n=15) and non-demented controls (n=17). **Table S2**. List of all 151 proteins found altered in SD dentate gyrus tissues, following differential abundance analysis. **Table S3**. Enrichment of upregulated proteins (n=131) for all three main GO categories. **Table S4**. Enrichment of downregulated proteins (n=20) for all three main GO categories. **Table S5**. SynGO enrichment analysis results for all 151 proteins found significantly altered in SD patients. **Table S6**. Protein-protein interactions extracted from STRING of all proteins with differential abundance in SD (n=151). **Table S7**. List of proteomic studies performed in FTLD (n=3) and AD (n=5) cohorts, selected for comparative analysis. **Table S8**. Comparison of proteins with differential abundance in SD to those identified previously in FTLD/AD proteomic datasets. **Table S9**. Quantified signals of immunoblots of three selected proteins. **Table S10**. First shell protein-protein interactions of the queried protein TARDBP (encoding for TDP-43) extracted from STRING.

**Additional file 2**. **Fig. S1**. Principal component analysis on peptide level. **Fig. S2**. Coefficient of variation (CV) on peptide and protein level. **Fig. S3**. SynGO enrichment analysis on all 151 deregulated proteins in SD. **Fig. S4**. Clusters of interacting proteins following PPI analysis. **Fig. S5**. Flowchart of comparative analysis. **Fig. S6**. Immunoblots of three selected proteins related to the cadherin-catenin complex. **Fig. S7**. Immunohistochemistry of N-cadherin in SD patients compared to controls.

## Data Availability

The mass spectrometry proteomics data have been deposited to the ProteomeXchange Consortium via the PRIDE partner repository [59] with the dataset identifier PXD033060.
