# Supplementary material for: Proteomics of the dentate gyrus reveals semantic dementia specific molecular pathology
Source: Acta Neuropathol Commun. 2022 Dec 28;10:190. doi: 10.1186/s40478-022-01499-1 (PMC9795759; doi:10.1186/s40478-022-01499-1)
Supplement: Supplementary file 1 — Additional file 1. Table S1. List of all quantified proteins following mass spectrometry of the dentate gyrus in SD patients (n=15) and non-demented controls (n=17). Table S2. List of all 151 proteins found altered in SD dentate gyrus tissues, following differential abundance analysis. Table S3. Enrichment of upregulated proteins (n=131) for all three main GO categories. Table S4. Enrichment of downregulated proteins (n=20) for all three main GO categories. Table S5. SynGO enrichment analysis results for all 151 proteins found significantly altered in SD patients. Table S6. Protein-protein interactions extracted from STRING of all proteins with differential abundance in SD (n=151). Table S7. List of proteomic studies performed in FTLD (n=3) and AD (n=5) cohorts, selected for comparative analysis. Table S8. Comparison of proteins with differential abundance in SD to those identified previously in FTLD/AD proteomic datasets. Table S9. Quantified signals of immunoblots of three selected proteins. Table S10. First shell protein-protein interactions of the queried protein TARDBP (encoding for TDP-43) extracted from STRING. [file 40478_2022_1499_MOESM1_ESM.pdf]

Supplementary Figures

- Figure S1. Principal component analysis on peptide level.
- Figure S2. Coefficient of variation (CV) on peptide and protein level.
- Figure S3. SynGO enrichment analysis on all 151 deregulated proteins in SD.
- Figure S4. Clusters of interacting proteins following PPI analysis.
- Figure S5. Flowchart of comparative analysis.
- Figure S6. Immunoblots of three selected proteins related to the cadherin-catenin complex.
- Figure S7. Immunohistochemistry of N-cadherin in SD patients compared to controls.

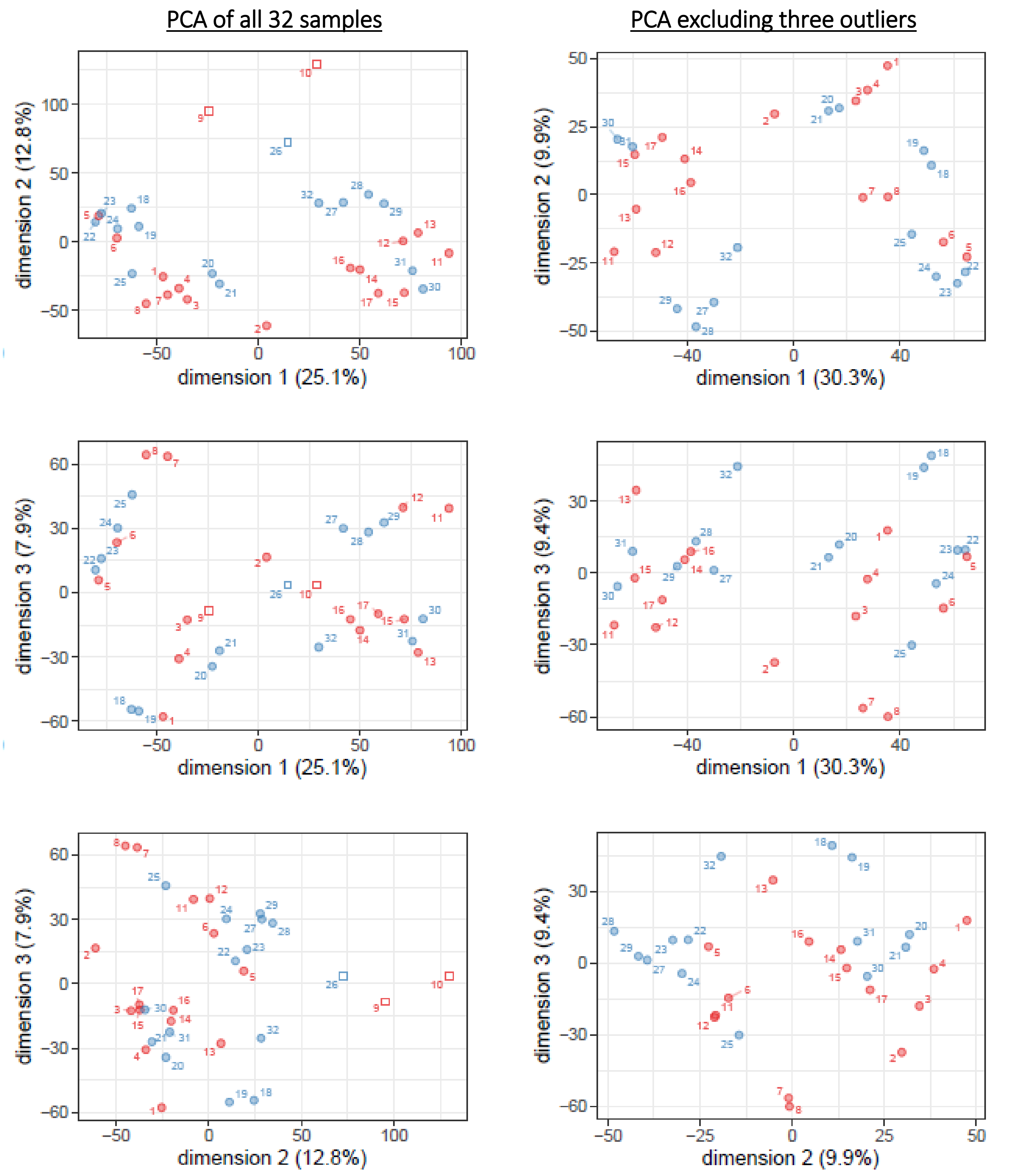

Figure S1. Principal component analysis on peptide level.

The pcaMethods R package was used to perform the Probabilistic PCA (PPCA) with all peptides passing the filter criteria. The figures on the left visualize the first three PCA dimensions of all samples (n=32), indicating three samples flagged as outliers (#9, 10, and 26; square shapes). The figures on the right visualize the first three PCA dimensions when excluding these three outliers. Samples are colored by experimental group, distinguishing controls (red) and SD patient samples (blue).

Peptide-level CV

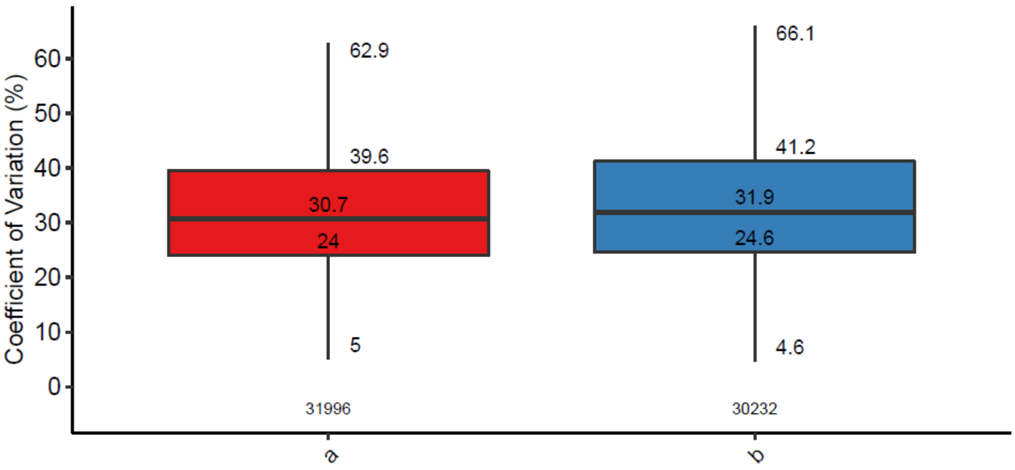

Protein-level CV

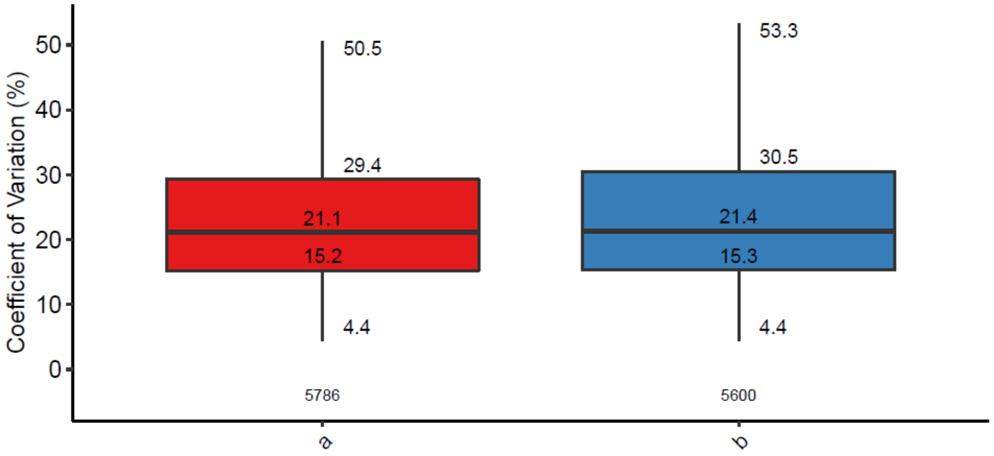

Figure S2. Coefficient of variation (CV) on peptide and protein level.

The coefficient of variation (CV) is a quality metric for the reproducibility of replicate measurements, here visualized using box -plots after removal of three sample outliers. This indicates that the precision of the assay was high, with a mean CV for peptide quantification of 31% for the control samples (group a) and 32% for the SD patients (group b). On protein level, mean CV was ~21% for both experimental groups. Note: only peptides with at least three data points across replicate samples were used for each CV computation.

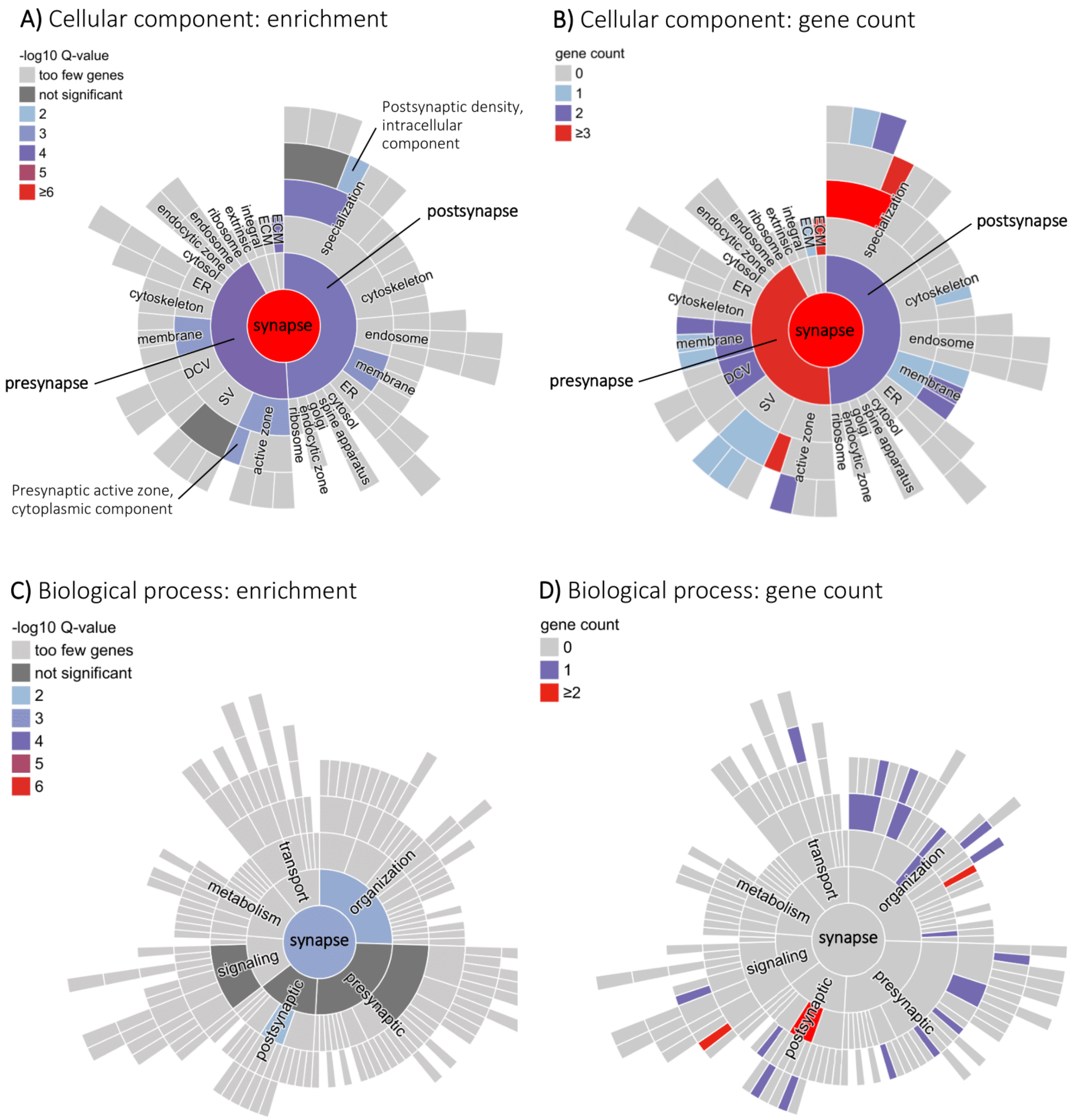

Figure S3. SynGO enrichment analysis on all 151 deregulated proteins in SD.

SynGO analysis was performed on cellular components (A,B) and biological processes (C,D) ontology terms, indicating synapse enriched proteins related to both presynaptic and postsynaptic compartments, as well as a range of synaptic functions. Sunburst plots are given both 'enrichment value' (-log10 Q-value) and 'gene count per term'. At 1% FDR threshold, enrichment analysis indicated 10 cellular components, including two more specific terms (third or fourth level in the hierarchical structure): 'presynaptic active zone, cytoplasmic component' and 'postsynaptic density, intracellular component'. For biological processes, three terms were significantly enriched. A complete overview including all terms, the hierarchical structure, and associated proteins is presented in Supplementary Table 5.

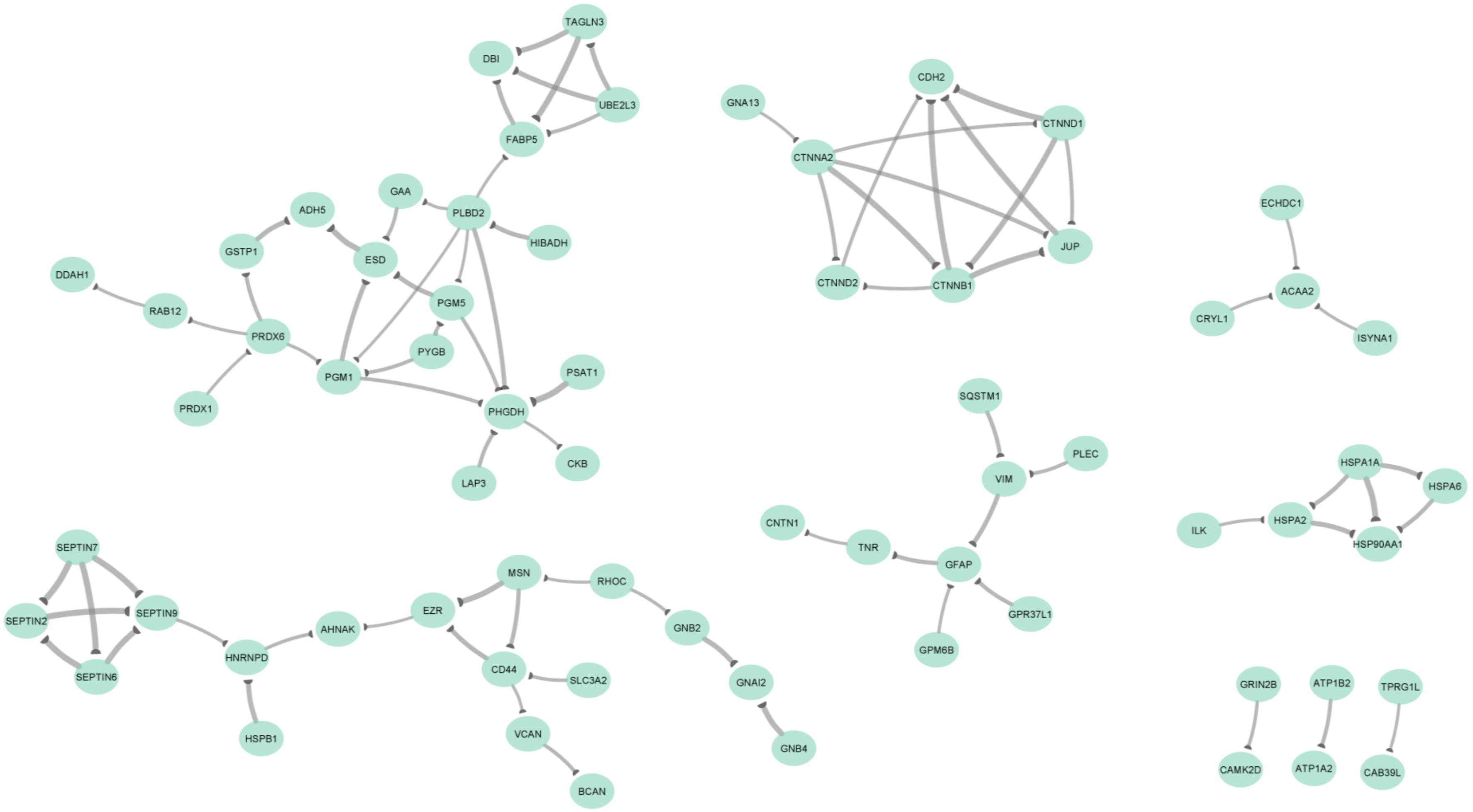

Figure S4. Clusters of interacting proteins following PPI analysis.

Amongst all 151 proteins with differential abundance in SD, we evaluated known protein-protein interactions using the STRING database. The interactions are based on phylogenetic co-occurence, co-expression, and experimentally determined interactions with a minimum combined score of 0.4 (medium confidence). In total, 68/151 proteins were found to interact with at least one other protein. The thickness of the edges corresponds to the combined interaction score. Further details are provided in Supplementary Table 6.

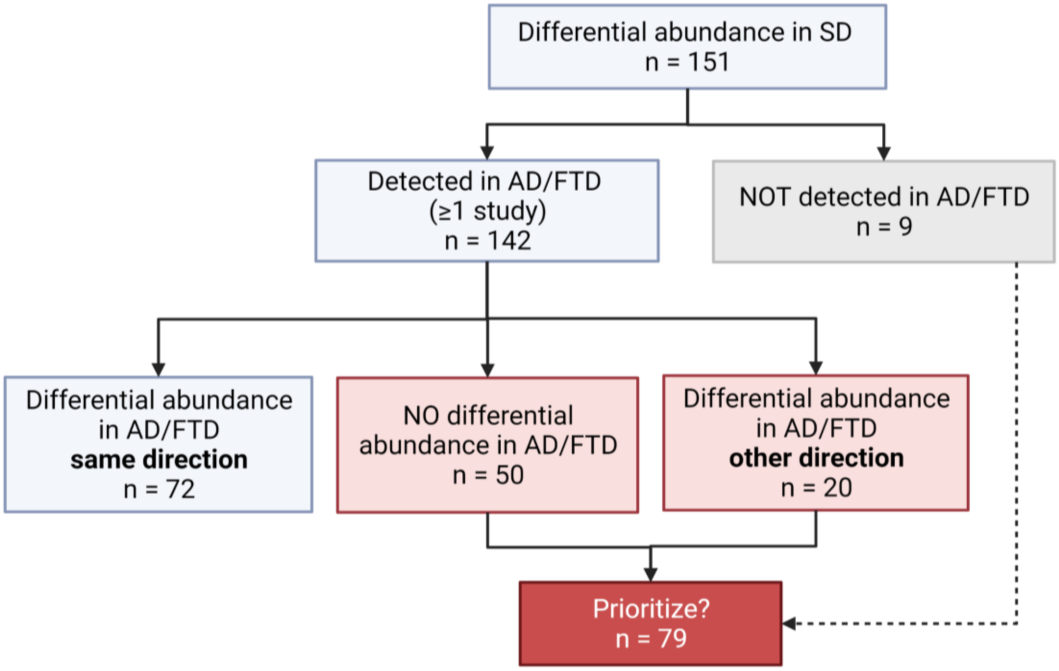

Figure S5. Flowchart of comparative analysis.

Protein abundances and statistical results of all proteins with differential abundance in SD were assessed in eight studies previously conducted in brain tissues of patients with Alzheimer's disease (AD) and frontotemporal dementia (FTD). A detailed overview of these studies is provided in Supplementary Table 7. Of the 151 unique proteins identified in SD, nine were not detected/quantified in the previous studies. Of the remaining proteins, 72 proteins showed the same direction of dysregulation in all disease types, implying alterations common to neurodegeneration. A total of 50 proteins were found altered in SD patients only, and 20 showed discordant differential abundance in AD/FTD. As such, these findings indicate 79 proteins with potentially unique profile of dysregulation in SD. A complete overview of all findings is presented in Supplementary Table 8.

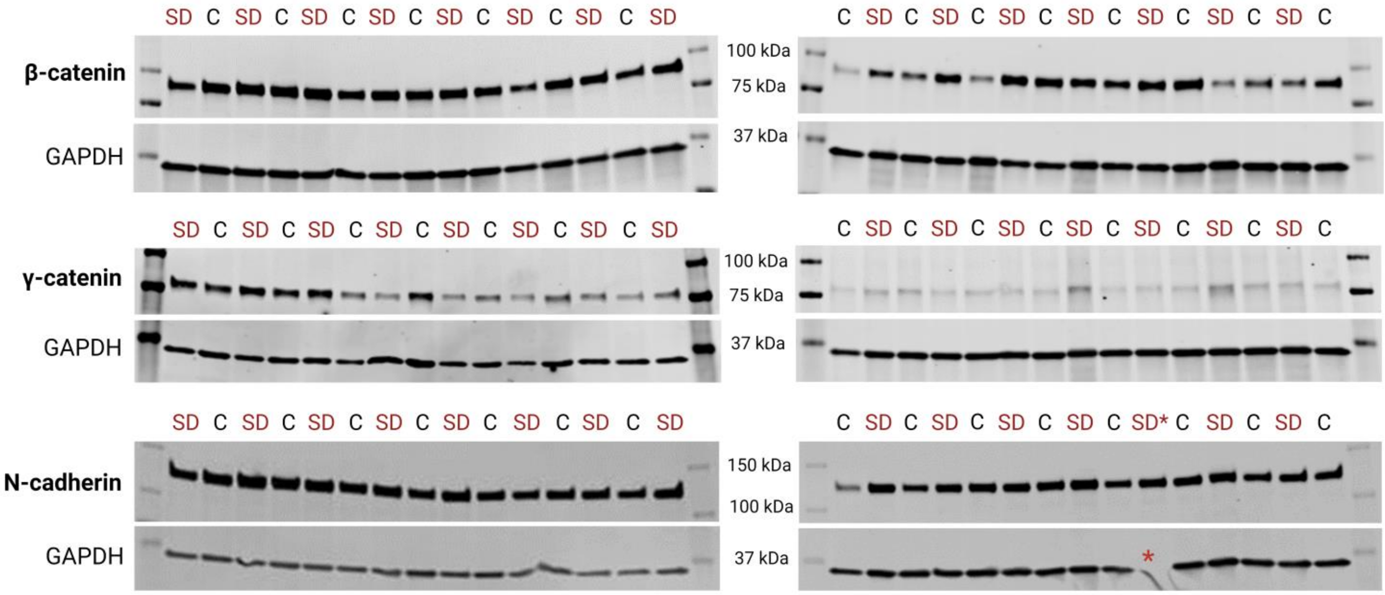

Figure S6. Immunoblots of three selected proteins related to the cadherin-catenin complex.

Protein extracts were prepared by lysis of whole hippocampal tissue (without centrifuging protein lysates) from 15 SD patients and 15 non-demented controls. Protein lysates were loaded on the gel in random order, with SD and control samples alternating. GAPDH was used as housekeeping gene for normalization of the quantified signals. The following primary antibodies were used: anti-β-catenin (1/1000, Santa Cruz, sc-7963), anti-γ-catenin (1/500, Cell signaling, #2309), and anti-N-cadherin (1/250, C32, BD Biosciences). Membranes were scanned using the Odyssey DLx system (LI-COR Bioscience), and the images quantified using Image Studio Lite software (version 2.0.38). The quantified data can be found in Supplementary Table 9. *In one case, GAPDH could not be ascertained; this sample was excluded from analysis.

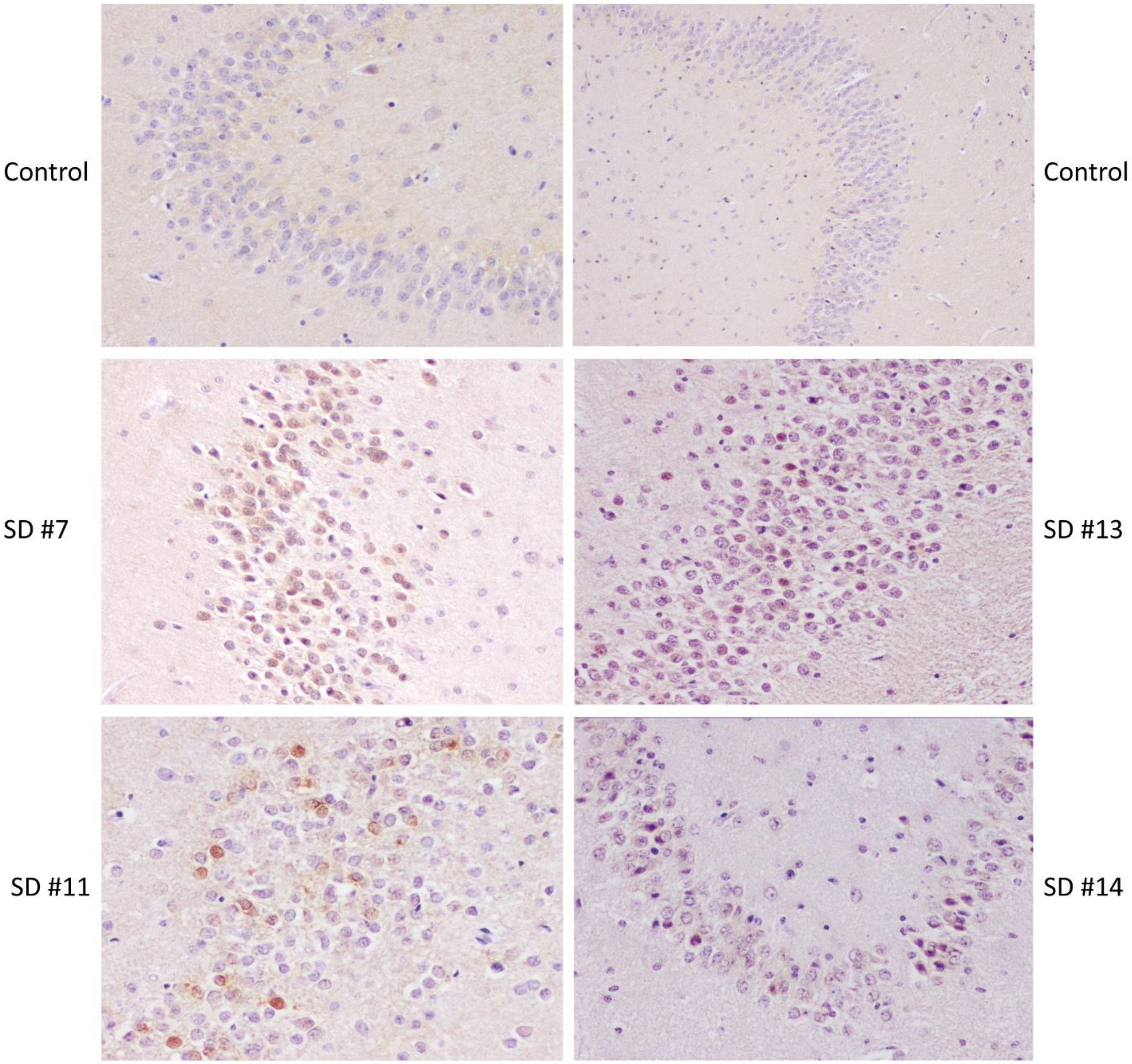

Figure S7. Immunohistochemistry of N-cadherin in SD patients compared controls.

Top row: staining of paraffin-embedded tissue with anti-N-cadherin resulted in diffuse background staining of the hippocampal dentate gyrus in non-demented controls.

Middle and lower row: We observed an irregular cytoplasmic staining pattern of the granular cells in four SD patients. This type of staining was seen in certain parts, but not throughout the whole dentate gyrus.
